# Life-long microbiome rejuvenation improves intestinal barrier function and inflammaging in mice

**DOI:** 10.1186/s40168-025-02089-8

**Published:** 2025-04-02

**Authors:** Felix Sommer, Joana P. Bernardes, Lena Best, Nina Sommer, Jacob Hamm, Berith Messner, Víctor A. López-Agudelo, Antonella Fazio, Georgios Marinos, A. Samer Kadibalban, Go Ito, Maren Falk-Paulsen, Christoph Kaleta, Philip Rosenstiel

**Affiliations:** 1 Institute of Clinical Molecular Biology, Christian-Albrechts-University and University Hospital Schleswig-Holstein, Kiel, 24105 Germany; 2 Institute of Experimental Medicine, Christian-Albrechts-University and University Hospital Schleswig-Holstein, Kiel, 24105 Germany; 3 Department of Gastroenterology, Gastrointestinal Oncology and Endocrinology, University Medical Center, Göttingen, Germany; 4 Department of Medicine I, University Medical Center Hamburg-Eppendorf, Hamburg, 20246 Germany; 5 CAU Innovation Gmbh, Christian-Albrechts-University, Kiel, 24118 Germany; 6 Department of Gastroenterology and Hepatology, Institute of Science Tokyo, Tokyo, Japan; 7 The Center for Personalized Medicine for Healthy Aging, Institute of Science Tokyo, Tokyo, Japan

**Keywords:** Aging, Microbiome, Rejuvenation, Lifespan extension

## Abstract

**Background:**

Alterations in the composition and function of the intestinal microbiota have been observed in organismal aging across a broad spectrum of animal phyla. Recent findings, which have been derived mostly in simple animal models, have even established a causal relationship between age-related microbial shifts and lifespan, suggesting microbiota-directed interventions as a potential tool to decelerate aging processes. To test whether a life-long microbiome rejuvenation strategy could delay or even prevent aging in non-ruminant mammals, we performed recurrent fecal microbial transfer (FMT) in mice throughout life. Transfer material was either derived from 8-week-old mice (young microbiome, yMB) or from animals of the same age as the recipients (isochronic microbiome, iMB) as control. Motor coordination and strength were analyzed by rotarod and grip strength tests, intestinal barrier function by serum LAL assay, transcriptional responses by single-cell RNA sequencing, and fecal microbial community properties by 16S rRNA gene profiling and metagenomics.

**Results:**

Colonization with yMB improved coordination and intestinal permeability compared to iMB. yMB encoded fewer pro-inflammatory factors and altered metabolic pathways favoring oxidative phosphorylation. Ecological interactions among bacteria in yMB were more antagonistic than in iMB implying more stable microbiome communities. Single-cell RNA sequencing analysis of intestinal mucosa revealed a salient shift of cellular phenotypes in the yMB group with markedly increased ATP synthesis and mitochondrial pathways as well as a decrease of age-dependent mesenchymal hallmark transcripts in enterocytes and TA cells, but reduced inflammatory signaling in macrophages.

**Conclusions:**

Taken together, we demonstrate that life-long and repeated transfer of microbiota material from young mice improved age-related processes including coordinative ability (rotarod), intestinal permeability, and both metabolic and inflammatory profiles mainly of macrophages but also of other immune cells.

Video Abstract

**Supplementary Information:**

The online version contains supplementary material available at 10.1186/s40168-025-02089-8.

## Introduction

**Fig. 1:**
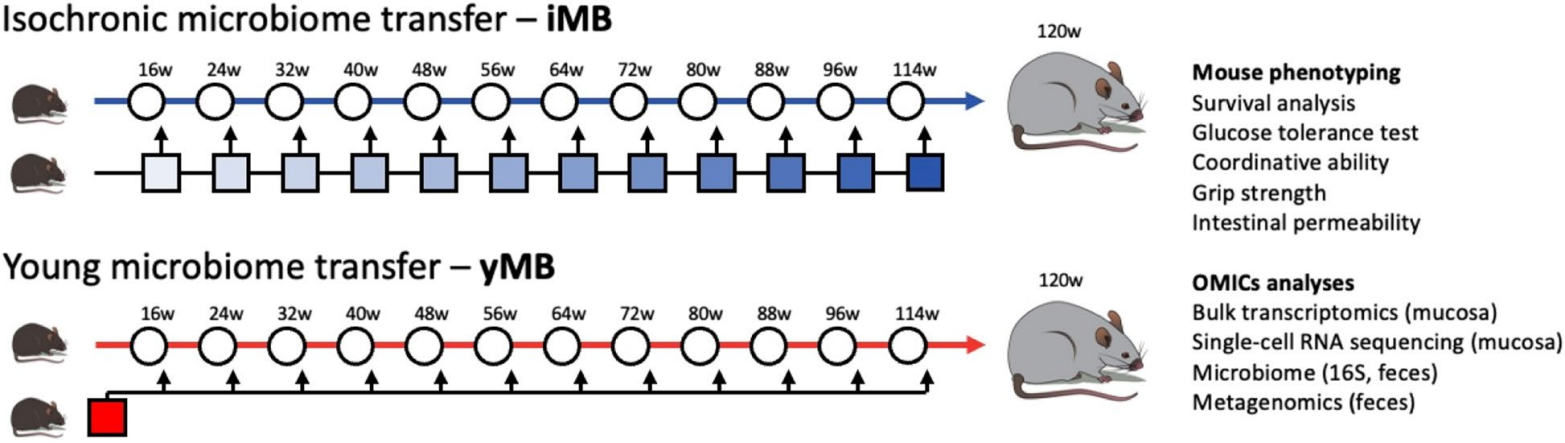
Schematic representation of microbial rejuvenation experiment. Eight-week-old male C57BL6/J wildtype mice (*n* = 40) were aged until 120 weeks of age and every 8 weeks mice (*n* = 20 per group) received a microbial transfer from unrelated wildtype mice being either of the same age as the recipients (isochronic microbiome transfer—iMB) or being 8-week-old (young microbiome transfer—yMB) with the goal to rejuvenate the aging recipient mice with a young microbiome. Mice were monitored throughout the experiment and extensively phenotyped at final analysis by functional and behavioral tests (rotarod, grip strength, intestinal barrier function, glucose homeostasis). Furthermore, colonic and small intestinal tissue were subjected to bulk and single-cell transcriptome sequencing, and fecal samples collected throughout the experiment were subjected to 16S rRNA amplicon sequencing and metagenomics

Organismal aging is accompanied by gradual physiological changes leading to a functional decline of almost all organ systems. There is evidence highlighting certain molecular hallmarks of aging such as genomic instability, telomere attrition, and mitochondrial dysfunction [1]. On the cellular level, senescence and stem cell exhaustion are among the major mechanisms that impair the functionality of organs and ultimately contribute to organismal death as an inevitable outcome of life [1]. Aging processes are not only determined by chronological time but also influenced by a complex interplay of genetic, epigenetic, environmental, and lifestyle factors [2]. While the passage of years indisputably plays a role in the aging of biological systems, the rate and manifestation of aging can vary significantly among individuals. Environmental factors including hygiene and nutrition also play a crucial role in shaping the trajectory of individual aging [3–5]. All these environmental factors impact on composition and function of the microbiome, the sum of associated microorganisms. Microbiome composition changes with age with conflicting reports of increased diversity in the elderly [6], possibly indicating a loss of control mechanisms, but also of reduced diversity [7–9], which is associated with numerous diseases [10]. Microbiota transfer experiments in killifish indeed demonstrated that the transfer of a young microbiome to middle-aged fish could extend lifespan [11]; however, direct proof for a causal role of aging-related microbiota changes on aging in mammals is limited. Two initial studies in mice revealed that transfer of an old microbiome in young mice increased the inflammatory tone [12] and that transfer of a microbiome from young to old mice could improve behavioral deficits and brain function [13–15], yet life-long interventions have not yet been attempted. We hypothesized that repeated transfers of a microbiome from young individuals throughout life may potentiate beneficial effects compared to a singular intervention and delay the organismal aging process. We hence conducted a microbiome intervention experiment by colonizing mice during their normal aging process recurrently every 8 weeks with either a fecal microbial transfer (FMT) of the young microbiome (yMB) or by FMT of unrelated mice of the same age (isochronic microbiome, iMB) throughout their lifespan (Fig. 1). We monitored the host’s physiological responses from an organismal down to a cellular level using single-cell RNA sequencing along with the microbial functional adaptations using fecal metagenomics. Our study not only provides evidence that a life-long intervention by FMT with intestinal microbiota from young mice interferes with features related to aging including intestinal barrier integrity and inflammaging, but also delivers a comprehensive overview of the molecular and functional changes in the intestinal mucosa and associated microbiome.

## Results

### Life-long microbiome rejuvenation affects physiological parameters and intestinal barrier function in mice

**Fig. 2:**
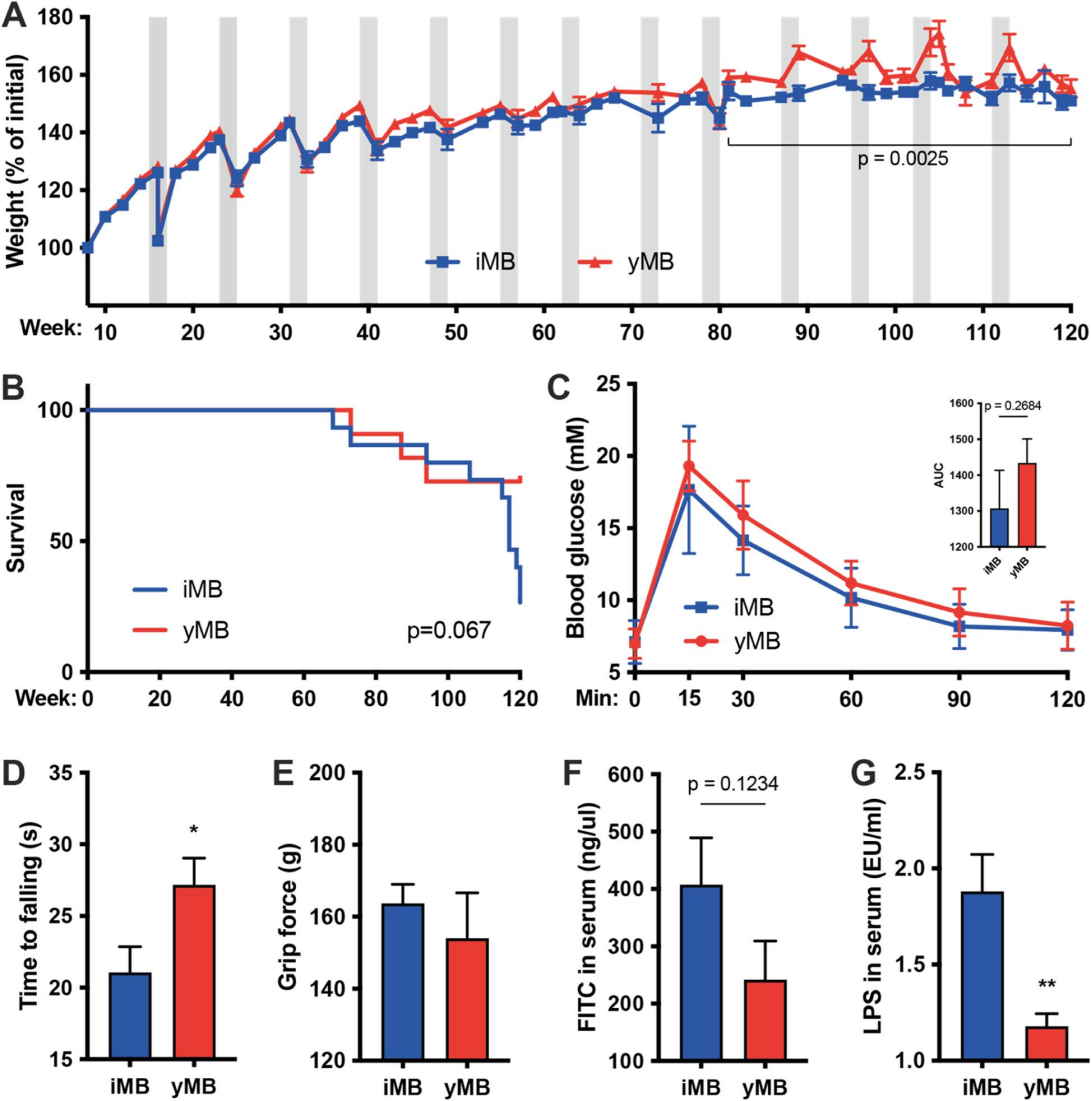
Microbial rejuvenation improves several host physiological traits.** A** Weight development. Grey areas denote phases of recurrent microbiome treatments. **B** Survival curve. **C** Blood glucose measurements during intraperitoneal glucose tolerance test (ipGTT) including area under the curve (AUC) summary. *n* = 5–6 per group. **D** yMB performed better in RotaRod performance test. *p < 0.05. *n* = 8–11 per group. **E** Grip strength as measured using a metal grid. *n* = 8–11 per group. **F** FITC dextran quantified in serum from vena cava 1 h after oral gavage. *n* = 5–6 per group. **G** Reduced LPS levels in vena cava serum of rejuvenated yMB mice. **p < 0.01. *n* = 7 per group

To test whether a microbiome rejuvenation strategy can improve or even delay aging-related features, we recurrently colonized mice during their normal aging process every 8 weeks with either a young microbiome (yMB) or with an isochronic microbiome (iMB) of unrelated mice of the same age (Fig. 1). Each colonization cycle consisted of a short antibiotic treatment to condition the intestinal tract for recolonization with the target microbiome. Fecal microbiome transfer was performed using oral gavage. During the first treatment cycles the mice expectedly experienced transient weight loss episodes [16–18] from which they quickly recovered once antibiotics were stopped. The transient weight loss diminished with each successive cycle, likely attributed to the animals acclimating to the experimental procedure. The animals continued gaining additional weight during physiological growth (Fig. 2A). Weight curves between groups remained similar until week 80 when yMB mice started displaying a higher body weight than iMB mice, which remained significant until the end of the experiment at week 120 (*p* = 0.0025 from Mann–Whitney test of the area under curve [AUC] of weeks 80–120). At week 120 the experiment had to be stopped as a large proportion of iMB mice quickly died (Fig. 2B) suggesting an extended lifespan in yMB mice although it did not reach formal statistical significance (*p* = 0.067 from Log-rank/Mantel-Cox test) due to the low number of remaining animals. At the end of the experiment, an intraperitoneal glucose tolerance test (ipGTT) was performed to monitor glucose homeostasis as alterations in glucose metabolism associated with aging in humans due to impairments in insulin secretion and action [19]. Our data clearly shows that microbiome rejuvenation did not alter glucose homeostasis in mice as blood glucose levels did not differ between yMB and iMB (*p* = 0.2684 from Mann–Whitney test of AUC, Fig. 2C) which is congruent with published reports noting differences between mice and human among glucose metabolism in aging [20]. To survey the coordinative ability and strength of the mice, a rotarod and a grip strength test were performed. yMB mice managed to remain longer on the rotating rod than iMB mice (*p* = 0.0385 from Mann–Whitney test, Fig. 2D), thus indicating that microbiome rejuvenation improved coordination. In contrast, the force required to detach mice from the grid did not differ between the treatment groups (*p* = 0.5999 from Mann–Whitney test, Fig. 2E), thus indicating that microbiome rejuvenation did not impact muscle function as assessed by grip strength. Finally, we analyzed intestinal barrier function, as leaking microbial antigens have been postulated to cause chronic low-grade inflammation and thereby contribute to the aging process [12, 13]. We orally gavaged FITC-labeled dextran and then quantified serum fluorescence. yMB mice trended towards lower serum FITC levels yet did not reach statistical significance due to low sample count (*p* = 0.1234 from Mann–Whitney test, Fig. 2F). Using the *Limulus* amebocyte lysate (LAL) assay we found that yMB had significantly reduced serum LPS levels compared to iMB mice (*p* = 0.0056 from Mann–Whitney test, Fig. 2G). These data indicate that microbiome rejuvenation improved intestinal barrier function leading to fewer leaked bacterial antigens.

### Restoration of metabolic activities in the rejuvenated microbiome

**Fig. 3:**
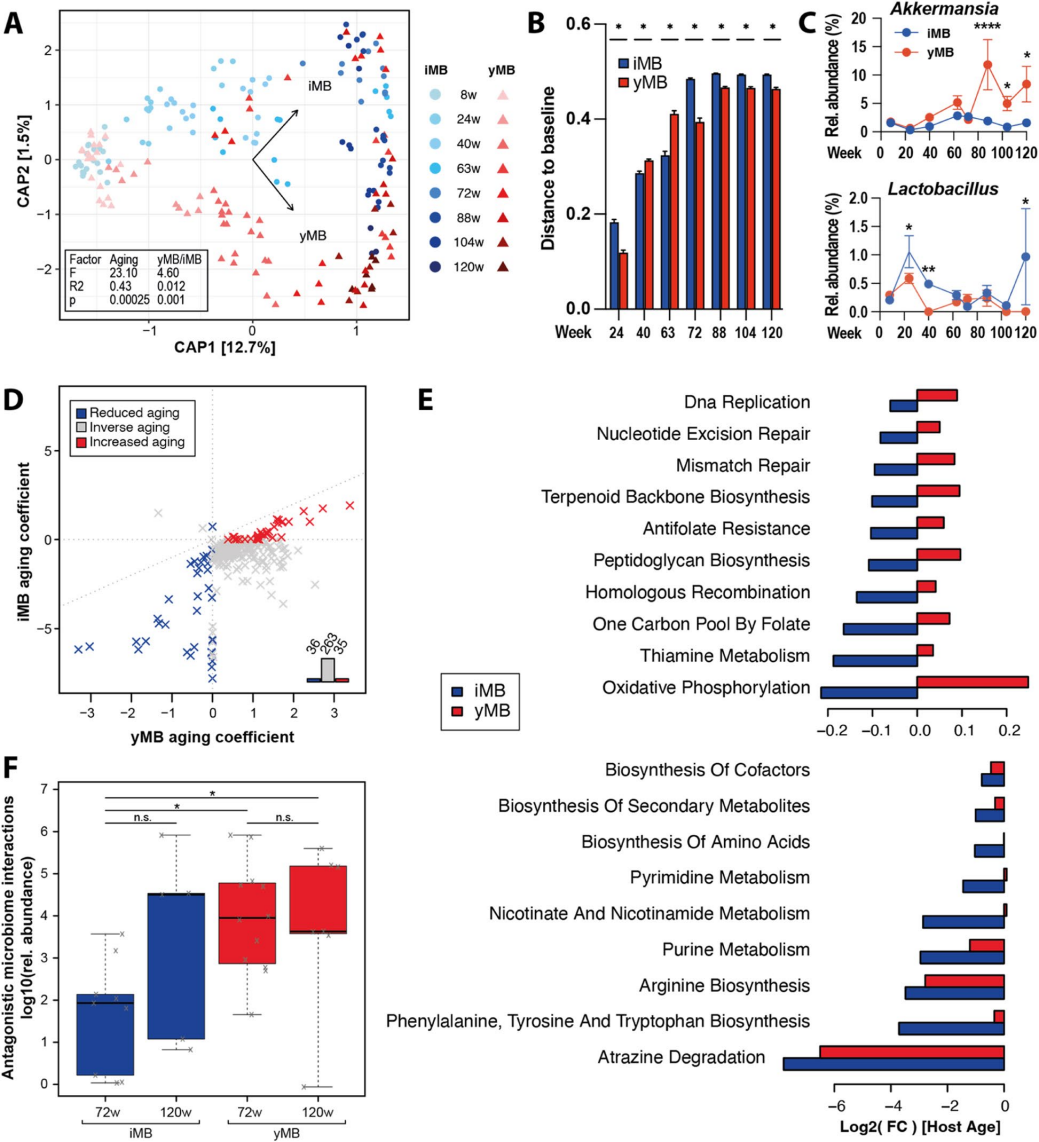
Microbiome rejuvenation reverts various age-associated microbial metabolic functions.** A** Constrained β-diversity plot of yMB and iMB microbiomes over time as determined by 16S rRNA gene amplicon sequencing. **B** Bray–Curtis distances from the baseline microbiome configuration (week 8) are smaller in yMB compared to iMB. A greater Bray–Curtis distance indicates more dissimilar microbiome compositions. **C** Relative abundance of the genera *Akkermansia* and *Lactobacillus*. **D** The majority of metagenome-derived metabolic functions (Humann3) had an inverted linear dependence on host age in yMB and iMB. **E** Pathway enrichment of those metabolic functions revealed distinct aging patterns for yMB and iMB. The aging effect in yMB is reduced, i.e. slowed down, in all enriched processes or even inverted for 12 out of 19 metabolic pathways. **F** Increased stabilizing antagonistic predicted microbe-microbe interactions in aged yMB compared to iMB mice

We next analyzed the temporal dynamics of the fecal microbiota throughout the rejuvenation experiment using 16S rRNA gene amplicon and metagenomic sequencing. Overall, the natural aging process of the host had the most significant impact on longitudinal microbiome changes in both treatment groups (Fig. 3A). However, the microbiome composition in the yMB intervention samples remained more similar to the initial young microbiomes (baseline, 8-weeks-old) than in iMB, as indicated by a lower Bray–Curtis distance, particularly at later timepoints (Fig. 3B). On a taxonomic level a few differences could be detected between yMB and iMB-treated mice, for example, for *Akkermansia* and *Lactobacillus*, both of which have been associated with healthy aging. The relative abundance of *Akkermansia* was increased whereas that of *Lactobacillus* was decreased in yMB compared to iMB-treated mice, especially toward the end of the experiment (Fig. 3C). Next, we inferred the adaptations in functional metabolic capacities of the microbial communities (KEGG orthologs) during aging and the rejuvenation intervention using the HMP Unified Metabolic Analysis Network (Humann3) from fecal metagenomic sequencing data. The effect of aging on microbiome reaction abundances differed in iMB as compared to yMB. Overall, 334 aging-dependent metabolic features (KEGG orthologs) were identified, of which the largest proportion (*n* = 263, ~ 80%) displayed an inverse age-dependent direction in yMB as compared to iMB, meaning upregulated features in yMB (old versus young) were found downregulated in iMB and vice versa. This indicates a reversal of age-related changes due to microbial rejuvenation (Fig. 3D, grey datapoints). The remaining metabolic features displayed concordant aging-dependent changes in both treatment groups with 35 features being upregulated in old versus young mice (Fig. 3C, red datapoints) and 36 being downregulated in both iMB and yMB (Fig. 3D, blue datapoints). Together, this indicates that age-dependent changes were less pronounced in yMB and thus a decelerated microbiome aging. An enrichment analysis among all age-associated metabolic features highlighted 19 bacterial pathways, all of which were more altered during aging in yMB mice compared to iMB mice (Fig. 3E and Table S1). Twelve bacterial pathways were positively associated with aging in yMB (i.e. showing a greater age-related change) but negatively associated with aging in iMB including, for example, peptidoglycan biosynthesis, nicotinate and nicotinamide metabolism, thiamine metabolism, and oxidative phosphorylation (Fig. 3E). Seven additional metabolic bacterial pathways such as purine metabolism, biosynthesis of cofactors or phenylalanine and tyrosine and tryptophan biosynthesis were negatively associated with aging but less pronounced in yMB (Fig. 3E and Table S1). Next, we modeled ecological relationships between pairs of bacterial species via flux balance analysis, as metabolite exchange is considered a keystone feature of a healthy and functional microbiome [21]. Antagonistic microbe-microbe interactions, which are considered a stabilizing factor for ecological communities [22–25], were significantly more abundant in the yMB-treated group than in iMB mice (Fig. 3F), thus hinting towards a more stable and interacting microbiome community in microbially rejuvenated mice, which are key features of a healthy host-microbiome ecosystem [26]. Altogether, these data therefore suggest that the rejuvenated microbiome in yMB mice provided beneficial metabolic functions to the host and thereby may contribute to delaying physiological processes associated with aging.

### Global transcriptional changes in the colon of rejuvenated mice

**Fig. 4:**
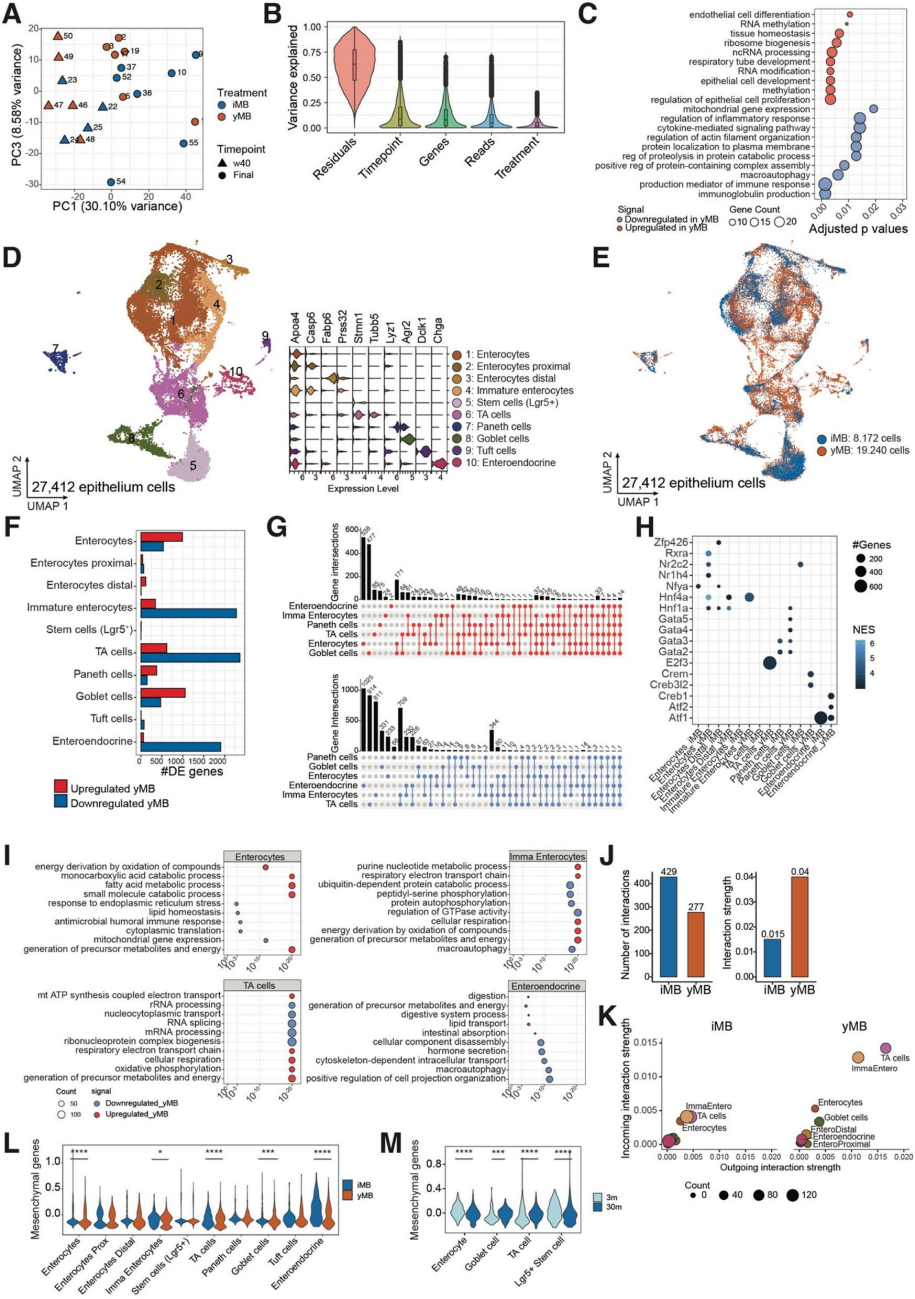
Transcriptional adaptations to the microbiome rejuvenation are specific to subtypes of intestinal epithelial cells. **A**–**C** Bulk RNA sequencing was performed on colon tissue of 40- and 120-week-old yMB and iMB mice. **A** Principal component plot. **B** Genes which expression changes contributed to the observed variance attributed to timepoint or treatment model. **C** Enriched functions among genes that were contributed to treatment explained variance. **D**–**L** Single-cell RNA sequencing of colonic samples of 120-week-old yMB and iMB mice. **D** Ten epithelial cell clusters were identified based on marker gene expression. **E** Treatment identity of all individual analyzed cells. **F** Number of up- and downregulated genes per epithelial cell type when comparing yMB vs iMB. **G** Shared and unique DEGs among epithelial cell types for up- and downregulated genes. **H** Transcriptional regulators enriched in upregulated DEGs. **I** Enriched functions for up- and downregulated genes (yMB vs iMB) in enterocytes, immature enterocytes, TA, and enteroendocrine cells. **J** Number and strength of predicted receptor-ligand interactions in yMB and iMB. **K** Outgoing and incoming ligand-receptor interaction strength of each epithelial cell type for iMB and yMB treatment. **L** Mesenchymal score reduced in yMB compared to iMB for multiple epithelial cell types indicating reduced EMT. **M** Reduced mesenchymal score in multiple colonic epithelial cells of 3- compared to 30-month-old mice of the *tabula muris senis* database [27, 28]

To get insights into the molecular changes in the intestinal mucosa, we performed bulk RNA sequencing of the ileum (Fig. S1) and colon (Fig. 4) tissue. Samples collected from 11 mice per treatment group were analyzed. We focused on two time points comprised of 40 weeks (40w) of age and the final time point of ~ 120 weeks (final). We first compared the transcriptional signatures between samples through a principal component analysis (PCA) and correlated them with the sample metadata (Fig. 4A and Fig. S1A). PC1 separated the two time points, 40 weeks and final, and explained 30.10% of the total variance. PC2 explained 12.95% of the total variance but it was driven mainly by the depth of the sample libraries, while PC3 was the component most related to the separation of the samples by treatment, explaining 8.57% of the total variance (Fig. S2). We calculated the gene expression changes that most contributed to variance using a variance partition analysis. Most variance was explained not by the model (outliers, timepoint, or treatment) but by the residuals (Fig. 4B and Fig. S1B). However, we identified 697 genes, whose expression was related to either yMB or iMB intervention independent of timepoints. We separated these genes into up- or downregulated in yMB versus iMB treatment and performed an enrichment analysis to identify the overarching molecular patterns characteristic of yMB intervention (Fig. 4C, Table S2). Within the top 25 enriched terms, we identified *mitochondrial gene expression, regulation of inflammatory response,* and *macroautophagy* among the downregulated terms of yMB, while *epithelial cell development*, *ribosome biogenesis*, and *lymphocyte proliferation* were characteristic of upregulated genes in yMB-treated mice. In summary, the analysis indicated profound changes in colonic gene expression along with the different treatment groups, which however may originate from different cellular composition or changes in cellular functional states.

### Cell type-specific responses to a young microbiome

We next aimed to map the global transcriptional effect of the microbiome rejuvenation intervention onto individual cell types using single-cell RNA sequencing. We used three mice from each yMB and iMB treatment group from the final timepoint (120 weeks). In total, we sequenced 53,658 cells isolated from the epithelial layer and lamina propria of the distal small intestine of the aged yMB and iMB mice with an average of 8943 cells per mouse. Of these 53,658 cells, 27,412 were classified as epithelial cells, 19,449 as immune cells, and 6797 as stromal cells. We firstly focused on the epithelial compartment, where we annotated 10 cell types based on *in-house* and reference-based markers ( [27, 28]—see “ Methods” section for details) (Fig. 4D). Both iMB and yMB treatments were represented in every cell type (Fig. 4E) and cell proportions did not differ significantly among treatments due to high interindividual variance (Fig. S3A). Shared and unique transcriptional states among epithelial cells of the iMB and yMB-treated mice were annotated and compared by a pairwise differential expression analysis to determine the differentially expressed genes (DEGs) characteristic of the yMB treatment for each individual cell type (Fig. 4F). Immature enterocytes, transit amplifying (TA) cells, and enteroendocrine cells displayed a higher number of downregulated DEGs in the yMB treatment, whereas enterocytes and goblet cells were characterized by a higher number of upregulated genes in the yMB treatment. Most of the DEGs were expressed by two or more cell types, but enterocytes had the most unique group of up- or downregulated genes (Fig. 4G). Next, we performed an enrichment analysis using the potential promoter sequences upstream of the signature DEGs (only upregulated genes) of each epithelial cell type aiming to identify significantly over-represented regulatory DNA motives and transcription factors that could be engaged by the microbiome rejuvenation (Fig. 4H). Among the yMB treatment, TFs that are essential for correct differentiation and repair of intestinal epithelium were identified in enterocytes (*Rxra*, *Nr1h4,* and *Hnf4a*), TA cells (*E2f3*), Paneth cells (*Gata4-5*) and enteroendocrine cells (*Creb1* and *Atf2*). We further expanded our analysis to both up- and downregulated genes in yMB and identified enriched GO terms of interest for each epithelial cell type (based on the top 25 terms sorted by lowest *p-*adjusted value—Fig. 4I, Table S2). Downregulated genes of yMB intervention from enterocytes were enriched for *mitochondrial gene expression* (multiple *Mrpl* genes, e.g., 11, 15, and 20) and *antimicrobial humoral immune response* (*Ccl25* and *Reg3b*) while the upregulated genes were enriched for *fatty acid metabolic process* (*Fabp1* and *Acsl1*). For immature enterocytes, the downregulated genes of yMB were enriched for *regulation of GTPase activity* and *macroautophagy* (*Traf6*, and *Atg* genes 12, 13, 2a, 2b, 4b, and 9a), while upregulated genes were enriched for *purine nucleotide metabolic process* (*Acaa2* and *Prdx5*). For TA cells downregulated genes of yMB treatment were involved in *mRNA* and *rRNA processing* (multiple *Rbm* genes, e.g., 10, 14, and 15b) while the upregulated genes were enriched also for *mitochondrial ATP synthesis* (multiple *Cox* genes, e.g., 4i1, 5a, and 5b). Finally, in enteroendocrine cells downregulated genes of yMB were enriched for *macroautophagy* (*Traf6*, and *Atg* genes 2b, 4d, and 16l2), and the upregulated genes were enriched for *digestive system process* and *lipid transport* (*Apoa1* and 4, and *Fabp1* and 2). Thus, differential functions identified in the bulk RNA sequencing data could be validated and delineated to single cell types. We further inferred ligand-receptor interaction differences between treatments by quantifying the signaling communication probability between paired cell groups using Cellchat [29]. For each treatment, we selected secreted and direct cell–cell contact signals to calculate the total number of interactions and their strength. This analysis identified an overall decrease in the number of interactions for the yMB intervention, yet the detected interactions were stronger (Fig. 4J) indicating a more focused transcriptional response after microbiome rejuvenation. All cell types contributed to the decrease in the number of interactions, but the increased strength of the observed interactions was mostly driven by immature enterocytes and TA cells (Fig. 4K). Finally, we investigated whether microbiome rejuvenation modulates the epithelial–mesenchymal transition (a process of transdifferentiation of epithelial cells that is linked to wound healing but also carcinogenesis) by testing the propensity of epithelial cells to express signature mesenchymal genes (*Vim*, *Ctnnb1*, *Fn1*, *Aifm2*, *Tgfb1*, *Tgfbr1,* and *Smad2-4*). Enterocytes, immature enterocytes, TA, goblet and enteroendocrine cells from the yMB intervention had significantly lower mesenchymal scores than iMB (Fig. 4L). Using the colon dataset of *Tabula Muris senis* we identified a significantly higher mesenchymal score in 30-month-old mice compared to 3-month-old mice (Fig. 4M), thus indicating that a transition to a more mesenchymal-like state is indeed a feature of old age, which could be reduced by our microbiome rejuvenation.

**Fig. 5:**
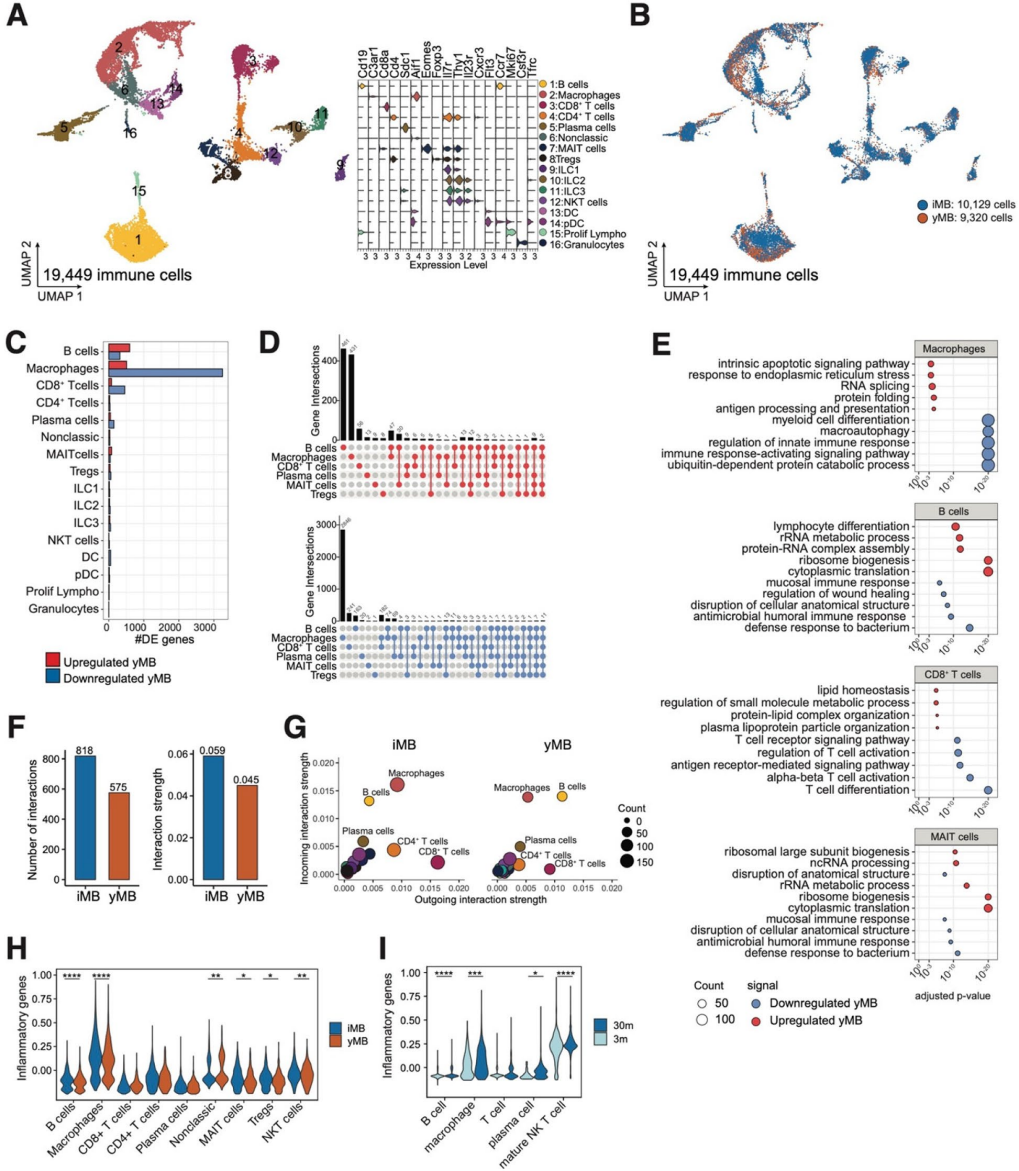
Macrophages are the main responding immune cell type to microbial rejuvenation. Single-cell RNA sequencing of small intestinal lamina propria immune cells of 120-week-old yMB and iMB mice. **A** 16 immune cell clusters were identified based on marker gene expression. **B** Treatment identity of all individual analyzed cells. **C** Number of up- and downregulated genes per immune cell type. **D** Shared and unique DEGs among immune cell types for up- and downregulated genes (yMB vs iMB). **E** Enriched functions for up- and downregulated genes (yMB vs iMB) in macrophages, B, CD8 + T, and MAIT cells. **F** Number and strength of predicted receptor-ligand interactions in yMB and iMB. **G** Outgoing and incoming ligand-receptor interaction strength of each immune cell type for iMB and yMB treatment. **H** Reduced inflammation score in yMB compared to iMB in multiple immune cell-types indicating a lower inflammatory tone. **I** Reduced inflammation score in splenic immune cells of 3- compared to 30-month-old mice of the *tabula muris senis* database [27, 28] indicating an increased inflammation score as a sign of aging

In addition to epithelial cells, we also queried the impact of microbial rejuvenation on the immune compartment. In total, 19,449 resident immune cells were identified, which were further annotated into 16 immune-specific cell types (Fig. 5A, see “ Methods” section for the annotation markers). As for epithelial cells, microbiome immune cell proportions did not differ significantly between yMB and iMB due to a high interindividual variation (Figs. 5B and 2B). Next, DEGs were calculated in yMB versus iMB treatments for each immune cell type (Fig. 5C, D). By far the most DEGs were identified in macrophages, with around 500 upregulated and 2900 downregulated genes in yMB (Fig. 5C), then B cells and CD8^+^ T cells followed by approximately 500 DEGs. In this case, the vast majority of DEGs were unique to single immune cell types (Fig. 5D). Enrichment analysis revealed that in macrophages *macroautophagy* (*Traf6,* and *Atg genes 12, 13, 2a, 4b 4c,* and *4d*) and immune processes such as *myeloid cell differentiation* and *regulation of innate immune response* (*Stat* genes *1, 3, 5a,* and *5b*) were underrepresented, whereas *antigen processing and presentation of exogenous peptide antigen *via* MHC class II, response to endoplasmic reticulum stress* and *intrinsic apoptotic signal* (Fcer1g, Atf6b, and Tora1) were enriched under yMB treatment (Fig. 5E, Table S2). Similar to epithelial cells, also in immune cells the number of ligand-receptor interactions was decreased in yMB, yet in contrast, the interaction strengths were also reduced (Fig. 5F). Mainly macrophages and T cells contributed to the decreased number and diminished strength of interactions (Fig. 5G). We aimed to query the immunomodulatory function of the microbiome intervention by creating a module score based on the expression of genes that are associated with inflammation during aging: *Tnf*, *Ifng*, *Il1b*, *Il2*, Il6*, Cxcl15*, *Ccl20*, *Ccl9*, *Ccr1*, *Nfkb1*, *Myd88*, and *Tlr6*. This inflammatory score was significantly reduced in multiple immune cell types in yMB versus iMB intervention including macrophages, B cells, MAIT, Tregs, and NKT cells (Fig. 5H). Notably, in the *Tabula muris senis* spleen data, the inflammatory score was similarly reduced in B cells, macrophages, and mature NK T cells in 3-month-old mice compared to 30-month-old mice (Fig. 5I), thus suggesting that the observed inflammatory score reduction in yMB indicates a rejuvenated profile.

## Discussion

Aging is a biologic process that is characterized by gradual molecular, cellular, and systemic changes leading to a decline of physiological functions in the organism over time [1]. Age-dependent changes in the intestinal microbiota have been linked to various physiological hallmarks of aging [12–15] and microbiome transfer experiments in fish even demonstrated a causal role in the aging process [11]. However, life-long interventions have not yet been attempted in higher mammals such as mice. In our study, we therefore aimed to slow down the aging process by employing a microbial intervention strategy that involved continuously introducing a young microbiome (yMB) into aging mice. We were particularly interested in how the microbiome would affect host physiology and intestinal cellular functions during aging. Microbial rejuvenation by recurrent yMB transfer improved several organismal physiological traits along with metabolic properties of the intestinal microbiome and transcriptional profiles of specific mucosal cell types, for example by boosting energy metabolism in enterocytes but blocking pro-inflammatory immune responses in macrophages. Continuous microbiome rejuvenation was able to delay physiological and molecular hallmarks of aging and ultimately improve organismal functions in mice.

### Microbial control of host cellular aging and immune response

Aging hallmarks can be classified into physiological functions (e.g., nutrient uptake, coordinative ability, or strength) and “molecular” features (e.g., reduced energy metabolic reactions, mitochondrial dysfunction, or improper immune responses). The intestinal microbiota has been linked to many of these processes. Our findings on intestinal barrier improvement and immune cell changes support previous research in which single colonization of young GF mice with a microbiome of old but not young SPF (specific-pathogen-free) mice sufficed to induce a proinflammatory environment [12]. Similarly, in another study, single colonization of young GF mice with a microbiome of old SPF donor mice induced defects in the intestinal barrier [13] making it leakier for microbial antigens—a physiological trait that under chronic conditions can lead to metabolic and inflammatory diseases. Therefore, the tightened intestinal barrier function in our yMB mice can be considered as a restored, healthier state [30], which is in line with reduced pro-inflammatory responses in the intestinal mucosa [31]. Coherent with the tightened intestinal barrier, the functional properties of the yMB group differed from iMB animals by presenting a more juvenile and beneficial profile with an increased abundance of the health-promoting bacterium *Akkermansia*, which has been shown to enhance health- and lifespan in mice [32] and to improve metabolic parameters in humans [33]. However, in yMB mice we found reduced levels of the genus Lactobacillus, although several studies indicated health-promoting effects of *Lactobacillus paracasei* HII01 [34] and *Lactobacillus plantarum* TWK10 [35]. These seemingly conflicting findings might be due to strain differences and the genus *Lactobacillus* encompasses many different strains. Furthermore, in line with the improved physiological parameters, the yMB microbiome encoded higher activities of folate and thiamine metabolism, which are established as being underrepresented in the elderly [36–39], but also lower levels of nicotinate/nicotinamide metabolism and tryptophan biosynthesis, which are two key metabolic pathways providing anti-inflammatory properties [40, 41] and their dysregulation has been linked to aging pathologies [42–45]. Further supporting a beneficial profile, cells of the intestinal mucosa of microbially-rejuvenated mice expressed higher levels of genes involved in purine nucleotide metabolism, and the yMB metagenome nucleotide metabolism pathways showed a “younger” profile with a reduced age association compared to iMB. Reduced microbial nucleotide production influences intestinal barrier function [46] and a drop of metabolic activity in an aging microbiome impairing host metabolic functions has recently been reported [47]. Of note, we observed in the bulk transcriptome data from colonic mucosa as well as in several cellular subpopulations a downregulation of a larger group of transcripts related to macroautophagy. This seems somewhat contradictory to previous findings which attributed a protective role of functional autophagy during healthy aging [48]. However, it must be considered that non-transcriptional mechanisms significantly contribute to autophagic flux [49], and therefore our observation of downregulated transcripts may not directly translate to an overall decrease in macroautophagy. Clearly, this finding warrants further investigation. Epithelial transdifferentiation into mesenchymal cells, also termed epithelial-mesenchymal transition (EMT), is a developmental process during the ontogeny of complex organisms [49]. Pathological EMT has been implicated in tumor cell invasion and metastasis, but also in non-malignant processes including aging, which coincides with fibrotic changes in many organ systems [50–53]. Indeed, we identified a high expression of mesenchymal genes in differentiating intestinal epithelial cell types of aging mice (both iMB and yMB), but remarkably in mice treated with the young microbiome mesenchymal gene expression was reduced.

Chronic, low-grade inflammation (inflammaging) is considered a key contributor to the gradual dysfunction of healthy physiological processes with increasing age. The process links to immunosenescent changes of immune cell compartments that typically comprise a sustained innate immune hyperreactivity whereas adaptive immune cells are increasingly compromised in their function [54–58]. In our study, the potential beneficial effects of yMB treatment align with the finding that the intestinal epithelium of rejuvenated mice exhibited molecular characteristics indicative of reduced pro-inflammatory immune responses. Among mucosal immune cells, intestinal macrophages showed the strongest differential response to the yMB intervention with the majority of genes (for example *Cd14*, *Myd88*, *Nod2*, and *Tlr1*, *2,* and *6*) being downregulated by the rejuvenating treatment. A composite inflammatory score based on the expression of known age-associated and proinflammatory mediators was reduced in various immune cell types in mice with a rejuvenated microbiome, thus adding further support to a reduced proinflammatory environment in the intestinal mucosa of yMB mice [59]. These aspects collectively underscore the benefits of microbiome rejuvenation in strengthening mucosal barrier integrity, reducing microbial antigen leakage, and suppressing processes related to inflammaging. Bacteria and other microbiome members have multiple means to affect host physiological processes, for example by producing metabolites that can be used by host cells to fuel metabolic reactions [47] or by exposing multiple antigens that trigger immune responses and train the immune system, which is important considering the concept of inflammaging [58]. For a comprehensive overview of these molecular interactions, we refer to other excellent reviews [1, 60–63]. Although our study highlights some molecular features, additional studies are required to disentangle the mechanistic framework of microbiome-host interactions.

### Improved mitochondrial function in microbially rejuvenated mice and impact on intestinal epithelial differentiation

As cells and organisms age, the efficiency of the respiratory chain declines, resulting in increased electron leakage and decreased ATP production [64]. Perturbed mitochondrial function in the intestinal mucosa has been shown to predispose to inflammation [65]. In rejuvenated mice, we found indications of improved mitochondrial function in several cell types within the intestinal mucosa. Mitochondria-dependent metabolic pathways such as monocarboxylic acid catabolism, fatty acid metabolism, oxidative phosphorylation, and respiratory electron transport chain activities were strongly boosted in enterocytes and TA cells of yMB compared to iMB mice. Moreover, the expression of mitochondrial ribosomal proteins was reduced in enterocytes of yMB mice. Notably, this group of evolutionary conserved genes has been linked to longevity and extended lifespan by inducing mitonuclear protein imbalance and an unfolded protein response [66]. Mitochondrial metabolism is associated with the production of reactive oxygen species (ROS), which play various roles in cellular functions such as pathogen defense. However, elevated ROS levels can also result in DNA damage, mutations, and malignant transformation [1]. Therefore, the increased metabolic activity of enterocytes and TA cells in yMB could point to both beneficial as well as detrimental processes in IEC. Notably, transcription factor enrichment analysis among the cell type-specific and yMB-dependent most upregulated genes highlighted several transcription factors that regulate differentiation and proliferation. For example, HNF4A, which controls epithelial cell differentiation and homeostasis [67], was overrepresented in yMB, whereas NFYA, which was underrepresented in yMB, has been linked to gastric adenocarcinomas [68] and aggressiveness of colorectal cancer [69]. ATF2, which was overtly present in yMB intervention, maintains epithelial regenerative capacity and protects against cell death during intestinal epithelial damage [70]. Taken together, these results highlight the potential microbial contribution to healthy aging by tuning mitochondrial function in a strongly proliferating cell type.

### Uniqueness of our study and potential limitations

Previous studies described physiologic rejuvenation upon single microbiome interventions in young mice [13–15]; however, these studies mainly employed germ-free mice, which have an altered immune system and intestinal mucosa with malfunctioning absorption and compromised permeability [10, 11]. Thus, we tried to circumvent this issue by using conventionally raised SPF mice and recurrently colonized them with either an isochronic or a young microbiome for their entire lifespan. We employed antibiotic preconditioning followed by repeated immediate seeding with yMB or iMB donor fecal material [26]. Previous studies have suggested that incorporating antibiotic therapy enhances efficacy in human FMT settings [71–73] by overcoming the resilience of the recipient microbiome and thereby improving engraftment, which is why we chose this approach in our proof-of-concept study. It must however be noted that schemes and effects of conditioning regimens vary widely in clinical studies, and the impact of repeated conditioning on engraftment and development of antibiotic resistance gene repertoires in mice remains to be clarified. At least in our study, we could not detect any differences in the emergence of antibiotic-resistance genes between yMB and iMB (Fig. S4). Nevertheless, we cannot rule out a potential confounding effect by the antibiotic cocktail, e.g., by shifting normal aging processes in both groups, which may hamper the generalizability of the finding to non-antibiotic treated animals. However, due to the identical treatment of both experimental groups, we consider this setup suitable to allow the conclusion that the observed phenotypic effects are specific to and caused by the yMB treatment. Due to the longitudinal study design, in which all animals in this cohort received treatment (antibiotics followed by either iMB or yMB), we were unable to reliably distinguish the residential “aged” microbiome from the donor microbiome. Future, larger-scale studies are needed to systematically compare the effects of gavage with solvent alone, antibiotic pre-treatment, and untreated aging controls to establish causal relationships between physiological changes and specific microbial taxa and/or functions.

We performed multiple OMICs analyses (bulk RNA sequencing, single-cell RNA sequencing, metagenomics) from small and large intestinal tissues and feces to thoroughly characterize the molecular adaptations during the microbiome rejuvenation experiment. However, due to technical problems, not all analyses are available from all tissues: the single-cell RNA sequencing failed from colon samples due to a high percentage of dead cells in the preparation and the *tabula muris senis* single-cell RNA sequencing database only contains data from colon tissue but not of small intestinal tissue. To account for this limitation, we performed bulk RNA sequencing from both small and large intestinal tissue, which revealed largely similar responses in both tissues.

Despite a relatively large initial number of animals (*n* = 20 per group) we narrowly missed the major endpoint of increased lifespan in the final part of the study. One potential explanation is the variability in the biological response to the treatment and the fact that several animals died in both groups at a relatively early timepoint, due to lesions introduced by the gavage. The study design itself may have contributed to the outcome; specifically, the dosing regimen of antibiotic treatments or the frequency of fecal microbiota transfers may not have been optimal for maximizing lifespan extension. We see it as a strength of our study, however, that we were able to detect significant changes in healthspan-related parameters (e.g., permeability and dampening of inflammation) that could precede measurable extensions in lifespan. Future pre-clinical studies should consider these variables, include a control group without antibiotic pre-conditioning, use less invasive FMT methods, and potentially incorporate a longer follow-up period.

## Conclusions

Continuous microbiome rejuvenation in mice sufficed to delay or even reverse several aging hallmarks including an improved coordinative ability, a tightened intestinal barrier, a dampened inflammatory tone, and altered metabolic profiles. The functional responses to microbiome rejuvenation vastly differed among cell types of the intestinal mucosa with, for example, boosted energy metabolism in enterocytes, but reduced proinflammatory responses in macrophages, indicating highly specific interactions between members of the intestinal microbiome and host cells. Altogether, our findings highlight the therapeutic potential of microbiome-based interventions to delay or alleviate aging-related pathophysiologies and promote healthy aging.

## Methods

### Animals

All animal experiments were approved by the local animal safety review board of the federal ministry of Schleswig Holstein and conducted according to national and international laws and policies (V 312–72,241.121–33 (95–8/11) and V242-62,324/2016 (97–8/16)). C57Bl6/J mice were purchased from Charles River Laboratories and housed in the Central Animal Facility (ZTH) of the University Hospital Schleswig Holstein (UKSH, Kiel, Germany) under specific-pathogen-free (SPF) conditions. All mice were kept under a 12-h light cycle and fed a regular gamma-irradiated chow diet ad libitum.

### Microbiome rejuvenation

Eight-week-old male C57Bl6/J mice (*n* = 60) were subjected to successive 8-week-long cycles consisting of (i) a microbiome depletion for 14 days via antibiotic treatment, (ii) immediate recolonization via fecal microbiota transfer and (iii) 6 weeks of an intermittent phase. To deplete the microbiome, mice were administered a cocktail of broad-spectrum antibiotics composed of ampicillin (1 g/L), vancomycin (500 mg/L), neomycin (1 g/L) and metronidazole (1 g/L) (Sigma Aldrich) [74, 75] ad libitum via drinking water in light protected bottles. Success of microbiome extinctions was controlled by DNA extraction from fecal pellets (see below) followed by real-time PCR amplification using 16S rRNA gene universal primers (F: ACT CCT ACG GGA GGC AG, R: GAC TAC CAG GGT ATC TAA TCC) and probe (CAG CAG CCG CGG TA) that target the V3–V4 region of the bacterial 16S rRNA gene. qPCR was performed in duplicate on a VIIA 7 PCR system (ThermoFisher, Waltham, MA) [76, 77]. Colonization by fecal microbiota transfer was performed as described previously [77, 78]. Briefly, freshly collected fecal pellets of unrelated and untreated male C57Bl6/J mice of the same age (isochronic microbiome transfer, iMB) or 8 weeks of age (young microbiome transfer, yMB) were resuspended in sterile PBS (~ 75 mg fecal material per ml PBS) and recipient antibiotics-treated mice were orally gavaged with 200 µl of the freshly prepared suspension to restore the depleted microbiomes. Up to five recipient mice were co-housed in individually ventilated cages (Green Line, Techniplast). Mice were weighed every 2 weeks and fecal pellets were collected before and after every antibiotic treatment, immediately frozen on dry ice, and stored at − 80 °C. A first batch of mice was killed by cervical dislocation and analyzed at 40 weeks of age, whereas all remaining mice were kept in the experiment until 122 weeks of age or until they had to be sacrificed for ethical reasons. Glucose tolerance was tested the week before sacrifice using an established protocol [78, 79]. Briefly, after fasting for 4 h, mice were i.p. injected with 20% D-glucose (2 g/kg body weight) and blood was drawn from the tail vein at 30, 0, 15, 30, 60, 90, and 120 min to measure blood glucose levels using an Accu-Chek Inform II glucometer (Roche). After a short recovery and washout phase, intestinal permeability was measured as described below and blood was collected from the vena cava, mice were killed and tissues were removed for histological and molecular analyses.

### Intestinal permeability assayin vivo

Intestinal permeability was quantified in the blood of mice using two parallel methods—FITC dextran gavage and HEK-Blue-TLR4 reporter assay—using established protocols as described before [76]. Briefly, mice of the microbiome rejuvenation experiment were fasted for 4 h and orally gavaged with 4 kDa FITC dextran (Sigma–Aldrich, 60 mg/100 g body weight). After 1 h mice were euthanized and blood was collected into gel-containing tubes. The serum was isolated by centrifugation at 10,000 × *g* for 5 min and diluted 1:1 with PBS. Fluorescence was measured on a spectrophotometer in 96-well plates at 528 nm. After background subtraction, FITC dextran concentrations were calculated using a standard curve prepared in PBS ranging from 0 to 800 μg/mL 4-kDa FITC dextran. In parallel, LPS levels were quantified in the blood of the yMB and iMB-treated mice using a HEK-Blue-TLR4 reporter cell line (Invivogen, hkb-htlr4) assay was used to measure the presence of LPS released in the serum. Cells were maintained according to the manufacturer’s protocol. Ten microliters of serum from yMB and iMB-treated mice were added to a flat bottom of a 96-well plate. HEK cells (140,000 cells/ml) resuspended in HEK-Blue detection medium (Invivogen) were added to each well. After overnight incubation at 37 °C in 5% CO_2_, plates were read on an Infinite M200 Pro Microplate Reader spectrophotometer (Tecan) at 620 nm and LPS levels were calculated using a standard curve of purified LPS according to the manufacturer’s protocol.

### Bulk RNA sequencing

Total RNA was extracted from colon biopsies using the RNeasy Mini Kit (Qiagen) according to the manufacturer’s protocol. RNA concentration and integrity were analyzed using a TapeStation 4200 System (Agilent) and a Qubit 4 fluorometer (ThermoFisher Scientific). RNA libraries were prepared using TruSeq stranded mRNA Kit (Illumina) according to the manufacturer’s instructions. All samples were sequenced using an Illumina NovaSeq6000 sequencer (Illumina, San Diego, CA, USA) with an average of 23 million paired-end reads (2 × 50 bp) at IKMB NGS core facilities. We used Tophat 2 [4] and Bowtie 2 [5] to align reads. Reads were mapped to the mouse genome (MGI assembly version 10) using Tophat 2 program. Expression counts were normalized by library size. Normalized gene expression values of the transcripts were computed by HTSeq [6]. To identify genes that were related to isochronic and young microbiome rejuvenation treatment differences we applied a variance partition approach using the variancePartition R package (v1.33.11). We further grouped these genes into gene ontology terms (GO) by performing a GO enrichment analysis for biological processes using the clusterProfiler package for R (version 4.10.1). To group the genes into up- or downregulated in the yMB intervention we used DEseq2 (v1.42.1) [7] to identify the differences in expression between the treatment groups based on their logfold change.

### Single-cell transcriptome sequencing

The small intestine was flushed with cold PBS, opened, and cut into two halves longitudinally. Intestinal epithelial cell (IEC) and lamina propria (LP) cell fractions were then isolated from one-half of the small intestine using the Lamina Propria Dissociation Kit (Miltenyi BioTech, Bergisch Gladbach, Germany) according to the manufacturer´s protocol with minor deviations as described before [8]. The composition of these cell fractions was analyzed by flow cytometry on a FACS Calibur flow cytometer (B&D, Heidelberg, Germany) with Cellquest analysis software (Becton Dickinson). The antibodies used are listed in Table S3. These IEC and LP cell fractions were immediately subjected to emulsion PCR using the 10 × Chromium system (10 × Genomics, Pleasanton, CA, USA) and the 10 × Chromium Single Cell gene expression V3 reagent kit according to the manufacturer’s instructions. Approximately 15,000 cells per sample were loaded. Eleven libraries were pooled and then sequenced on an Illumina NovaSeq6000 with 2 × 100 bp on an S2 flow. Sequences were mapped using cell ranger (v3.0.2) to the GRCm38 *Mus musculus* genome reference. We used the unfiltered feature-barcode matrices and applied custom filter settings under Seurat package environment (v3.1.2). Cells that were potentially disrupted or had an increased likelihood of being doublets (cells with less than 200 or more than 5000 reads mapping to genome, with more than 25% of mitochondrial reads or less than 10% of housekeeping reads), were removed according to best practices [80]. Doublet detection was performed using DoubletFinder R package (v2.0.2). IEC and LP objects were merged per mouse sample, then all samples were integrated per mouse and into a single object containing both iMB and yMB groups. The merged object was analyzed using standard Seurat workflow [81]. We used all genes to scale the data, identified the top 2000 variable features for the principal component analysis, used the top 80 dimensions for finding neighbors, and ran the dimensionality reduction (UMAP). Clusters were identified using a shared nearest neighbor (SNN) modularity optimization clustering algorithm with a resolution of 0.2 [82]. Clusters were classified into compartments based on the expression of prototypical marker genes (epithelial cells: *Epcam, Krt8, Krt18*; stromal cells: *Col1a1, Col1a2 Col6a1, Col6a2, Vwf, Plvap, Cdh5, S100b*; immune cells: *Cd52, Cd2, Cd3d, Cd3g, Cd3e, Cd79a, Cd79b, Cd14, Fcgr3, Cd68, Cd83, Csf1r, Fcer1g*). Epithelial cells were further classified into individual IEC subtypes using biomarkers previously described in published literature [83]: enterocytes (*Apoa4*), proximal enterocytes (*Apoa4* and *Casp6*), enterocytes distal (*Apoa4* and *Fabp6*), immature enterocytes (*Apoa4* and *Prss32*), stem cells Lgr5^+^ (*Stmn1*), Transit Amplifying (TA) cells (*Stmn1* and *Tubb5*), Paneth cells (*Lyz1*), goblet cells (*Agr2*), tuft cells (*Dclk1*) and enteroendocrine cells (*Chga*). Immune cells were further classified to cell types using the SingleR package (version 1.0.6) (Aran et al., 2019) and biomarkers: B cells (*Cd19*), Macrophages (*C3ar1*), CD8^+^ T cells (*Cd8a*), Cd4^+^ T cells (*Cd4*), plasma cells (*Sdc1*), non-classic myeloid cells (*Aif1*), MAIT cells (*Eomes*), Treg cells (*Foxp3*), ILC1 (*Il7r*), ILC2 (*Il7r* and *Th1*), ILC3 (*Il7r* and *Il23r*), NKT cells (*Cxcr3*), Dendritic cells (DC) (*Flt3*), plasma DC (pDC) (*Ccr7*), Proliferative lymphocytes (*Mki67*), and granulocytes (*Csf3r*). We calculated the cell type proportion for each sample of mice, we tested the differences in cell proportions between treatments by a Mann–Whitney test for non-parametric data. Differentially expressed genes (DEGs) between yMB and iMB treatments were identified per cell type using a Wilcoxon Rank Sum test with a log-fold difference of 0.1 between the two treatments (adjusted *p* value < 0.05 based on Bonferroni correction using all genes in the dataset). We used these genes to perform a GO enrichment analysis for biological processes using the clusterProfiler package for R (version 4.10.1) [83]. The most interesting GO terms from the top 25 GO list for both downregulated and upregulated genes under yMB treatment were plotted by cell type. Furthermore, we calculated the upregulated genes for each combination of cell type and treatment to do a transcription factor enrichment analysis using RcisTarget for R package (version 1.20.0). Finally, we used CellChat package for R (version 2.1.2) to identify the ligand-receptor pairs that were enriched for one of the treatments and which cell types of ligand-receptor pairs were most impacted by the treatments. To test whether our findings might represent age-related features, we performed a parallel analysis using the large intestinal subsets of *Tabula Muris Senis* [27, 28]. Similarly to our study, we used their annotated epithelial cell types and further annotated the missing (e.g., “nan” as enteroendocrine cells based on the markers described above) and tested whether the stromal gene module and the inflammatory score module showed a difference between young (3-month-old mice) and aged mice (30-month-old mice) in the epithelial compartment of large intestine and spleen of *Tabula Muris Senis* dataset.

### Microbiome analysis using 16S rRNA gene amplicon sequencing

DNA was isolated from fecal material using the DNeasy PowerSoil Kit (Qiagen) following the manufacturer’s protocol. Extracted DNA was eluted from the spin filter silica membrane with 100 µl of elution buffer and stored at − 80 °C. 16S rRNA gene amplicon profiling and MiSeq sequencing were performed as described earlier [84, 85], with the following modifications: the V3–V4 region of the 16S rRNA gene was amplified using the dual barcoded primers 319F (ACT CCT ACG GGA GGC AGC AG) and 806R (GGA CTA CHV GGG TWT CTA AT) [86]. Each primer contained additional sequences for a 12-base Golay barcode, Illumina adaptor, and a linker sequence [87]. PCR was performed using the Phusion Hot Start Flex 2X Master Mix (NEB) in a GeneAmp PCR system 9700 (Applied Biosystems) and the following program (98 °C for 3 min, 25–30 × [98 °C for 20 s, 55 °C for 30 s, 72 °C for 45 s], 72 °C for 10 min, hold at 4 °C). The performance of the PCR reactions was checked using agarose gel electrophoresis. Normalization was performed using the SequalPrep Normalization Plate Kit (Thermo Fisher Scientific, Darmstadt, Germany) following the manufacturer’s instructions. Equal volumes of SequalPrep-normalized amplicons were pooled and sequenced on an Illumina MiSeq (2 × 300 nt). MiSeq sequence data was first subjected to quality control and sample mapping using MacQIIME v1.9.1 (http://www.wernerlab.org/software/macqiime). Briefly, sequencing reads were trimmed keeping only nucleotides with a Phred quality score of at least 20, then paired-end assembled and mapped onto the different samples using the barcode information. The sample-mapped MiSeq 16S rRNA gene amplicon sequence data were then further processed using DADA2 [88] workflow (https://benjjneb.github.io/dada2/ bigdata.html) with default parameters resulting in abundance tables of amplicon sequence variants (ASVs). Taxonomy was assigned using the Bayesian classifier provided in DADA2 and using the Silva rRNA database v.138 [89]. Uni- and multivariate analyses of the 16S rRNA gene amplicon data were done in R (v.4.2.1) under phyloseq [90] (v.1.40.0), vegan [91] (v.2.6–2) and MAasLin2 [92] (v.1.10.0). All samples for diversity analyses were normalized by rarefaction to the minimum shared read count to account for differential sequencing depth among samples. Relative abundance was calculated by dividing the number of reads for an ASV by the total number of sequences in the sample. Beta diversity was computed using Bray–Curtis and differences were visualized after a constraint analysis of principal coordinates based on Bray–Curtis distances. To detect differences in changes in microbial features between yMB and iMB over time or among mucosal or luminal small intestine or colon tissue, we built linear mixed models using the MaAslin2 package [92] in previously wrench normalized abundances [93]. The model included time or/and a treatment group, and individual mice as a random variable. *p* values were corrected for multiple hypothesis testing using the Benjamin-Hochberg procedure, and a false discovery rate < 0.05 was defined as the significant threshold. Only features appearing in at least 20% of the samples were included in the analysis.

### Microbiome analysis using 16S rRNA gene amplicon sequencing

DNA was isolated from fecal material using the DNeasy PowerSoil Kit (Qiagen) following the manufacturer’s protocol. Extracted DNA was eluted from the spin filter silica membrane with 100 µl of elution buffer and stored at − 80 °C. 16S rRNA gene amplicon profiling and MiSeq sequencing were performed as described earlier [84, 85], with the following modifications: the V3–V4 region of the 16S rRNA gene was amplified using the dual barcoded primers 319F (ACT CCT ACG GGA GGC AGC AG) and 806R (GGA CTA CHV GGG TWT CTA AT) [86]. Each primer contained additional sequences for a 12-base Golay barcode, Illumina adaptor, and a linker sequence [87]. PCR was performed using the Phusion Hot Start Flex 2X Master Mix (NEB) in a GeneAmp PCR system 9700 (Applied Biosystems) and the following program (98 °C for 3 min, 25–30 × [98 °C for 20 s, 55 °C for 30 s, 72 °C for 45 s], 72 °C for 10 min, hold at 4 °C). Performance of the PCR reactions was checked using agarose gel electrophoresis. Normalization was performed using the SequalPrep Normalization Plate Kit (Thermo Fisher Scientific, Darmstadt, Germany) following the manufacturer’s instructions. Equal volumes of SequalPrep-normalized amplicons were pooled and sequenced on an Illumina MiSeq (2 × 300 nt). MiSeq sequence data was first subjected to quality control and sample mapping using MacQIIME v1.9.1 (http://www.wernerlab.org/software/macqiime). Briefly, sequencing reads were trimmed keeping only nucleotides with a Phred quality score of at least 20, then paired-end assembled and mapped onto the different samples using the barcode information. The sample-mapped MiSeq 16S rRNA gene amplicon sequence data were then further processed using DADA2 [88] workflow (https://benjjneb.github.io/dada2/ bigdata.html) with default parameters resulting in abundance tables of amplicon sequence variants (ASVs). Taxonomy was assigned using the Bayesian classifier provided in DADA2 and using the Silva rRNA database v.138 [89]. Uni- and multivariate analyses of the 16S rRNA gene amplicon data were done in R (v.4.2.1) under phyloseq [90] (v.1.40.0), vegan [91] (v.2.6–2) and MAasLin2 [92] (v.1.10.0). All samples for diversity analyses were normalized by rarefaction to the minimum shared read count to account for differential sequencing depth among samples. Relative abundance was calculated by dividing the number of reads for an ASV by the total number of sequences in the sample. Alpha diversity measures and beta diversity were computed using Bray–Curtis and differences were visualized after a constraint analysis of principal coordinates based on Bray–Curtis distances. To detect differences in changes in microbial features between yMB and iMB over time or among mucosal or luminal small intestine or colon tissue, we built linear mixed models using the MaAslin2 package [92] in previously wrench normalized abundances [93]. The model included time or/and a treatment group, and individual mice as a random variable. *p* values were corrected for multiple hypothesis testing using the Benjamin-Hochberg procedure, and a false discovery rate < 0.05 was defined as the significant threshold. Only features appearing in at least 20% of the samples were included in the analysis.

### Metagenomics

The fecal DNAs were subjected to shotgun metagenomic sequencing performed at the Competence Centre for Genomic Analysis (Kiel). DNA libraries were generated using the Illumina DNA Prep kit following the manufacturer’s instructions. Libraries were then pooled and sequenced on an Illumina NovaSeq 6000 with 2 × 150 nt for approximately 30,000,000 reads per sample. Raw reads were adapter trimmed using cutadapt (version 2.8) and quality trimmed to a mean PhredScore > = 30 using prinseq lite (version 0.20.4). Reads shorter than 50 bp were subsequently discarded. To remove host DNA contamination, only reads that did not map against the mouse reference genome (GRCm38.99, 2020–02–03) via HISAT2 (version 2.1.0) were kept for further analyses. For performance reasons, the remaining reads had to be downsampled to 30 million reads per library via Seqtk (version 1.3-r114-dirty), which resulted in (at most) 60 million reads per sample given paired-end sequencing with a forward and reverse read library each. The obtained metagenomic reads were used with the Humann3 pipeline (version 3.0.1) to generate KEGG ortholog count estimates per sample. Due to low mapping rates, 5 samples had to be removed from further metagenomic analyses leaving *n* = 9 samples for iMB 72 weeks, *n* = 5 for iMB 120 weeks, *n* = 12 for yMB 72 weeks, and *n* = 7 for yMB 120 weeks respectively.

### Prediction of ecological relationships

Interactions between members of the microbiome but also the host form the basis for many ecosystem properties including nutrient cycling and food webs. Here, the ecological interactions refer to the growth of a given bacterium of the microbiome in comparison to the entire microbiome. Metagenomic reads were mapped against a collection of 181 mouse fecal metagenomic assembled genomes (MAGs) and their respective metabolic models. This abundance data was then combined with the prediction of ecological relationships [94] between each pair of bacteria (MAG) as previously described [47] for each individual mouse microbiome community. Briefly, we employed flux balance analysis, a mathematical approach for studying metabolic networks built from all known reactions in an organism, to estimate the growth of each single bacterial model and compared this growth rate to that achieved by the models when co-grown in pairs of two different bacterial species. For more details on FBA, please see [47]. The six types of ecological relationships [94] and their frequencies among each microbial community were inferred with the R package EcoGS (https://github.com/maringos/EcoGS).

*Statistical analysis of microbiota metabolic functions and ecological relationships* KEGG ortholog count tables and ecological relationship abundances were subsequently analyzed with R. Only features (namely KEGG orthologs or ecological relationships) with abundance greater zero in at least 10% of the samples were considered for further analysis. Then the raw abundances were normalized sample-wise by the total sum scaling method. Relative KEGG ortholog abundances were transformed with the formula log10(*x* + 0.01) + 2, in order to keep zero counts at zero after log10 transformation while offsetting the non-zero values only minimally. Since ecological relation abundances were all non-zero, a simple log10 transformation was employed for those. For each feature, the abundance data was modeled as the dependent variable in a linear mixed-effects model with age, treatment, and their interaction term as fixed effects as well as with mouse identifier as random effect (function lmer from package lme4 version 1.1–29). Probability values were corrected for multiple testing via the method of Benjamini and Hochberg (function *p*.adjust with method = “BH”). Features with a significant false discovery rate (FDR ≤ 0.05) in the interaction term of age and treatment were further evaluated. The estimated regression coefficients of age for each treatment group were extracted from the linear mixed-effects models for each KEGG ortholog and plotted. Finally, significantly different KEGG orthologs were checked for their membership in KEGG pathways with function enricher (minGSSize = 3, maxGSSize = 500, pAdjustMethod = “BH”) of package clusterProfiler (version 4.4.1) and the KEGG pathway annotation database of the Humann3 software (version 3.0.1). As the enrichment analysis was based on only the significantly different KEGG orthologs as input, all pathways with a *p* value *p* ≤ 0.05 and with at least three features enriched for that pathway were reported to condense the gene results into a higher-level context (pathways). Log2 fold changes of pathway abundance were calculated between time-points 120 and 72 weeks for all features of an enriched pathway individually and then summarized via their arithmetic mean.

### Antibiotic resistance genes

Metagenomic reads were mapped against known antibiotic resistance genes obtained from the comprehensive antibiotic resistance database [95] (CARD, v3.1.2) using HISAT2 (version 2.1.0) followed by statistical analysis of read counts per antibiotic resistance (ABR) gene of the “protein_homolog” and “protein_overexpression” category in R. Statistical differences in read counts were tested for each ABR gene between treatment groups of the same age via Wilcoxon Rank Sum test followed by *p* value adjustment for multiple testing via the method of Benjamini and Hochberg. Additionally, the combined total read counts over all ABR genes and the combined read counts of the ABR genes within a resistance class were compared between treatment groups of the same age with the same methodology. No statistical significance was reached after correction for multiple testing via the Benjamini–Hochberg method.

### Statistical analysis

Biostatistical analyses were performed using GraphPad Prism (version 8) software (GraphPad, Inc, La Jolla, CA, USA), MacQIIME v1.9.2, or R (v 3.2.5). Specific comparisons and analyses are described in the individual method sections. Differences between the groups were considered significant at *p* < 0.05 and the data are presented as means ± SEM.

## Supplementary Information

Additional file 1. Fig S1. Bulk transcriptional adaptations to the microbiome rejuvenation in ileum tissue. Bulk RNA sequencing was performed on ileum tissue of 40- and 120-week-old yMB and iMB mice. A) Principal component plot. B) Genes which expression changes contributed to the observed variance attributed to timepoint or treatment model. C) Enriched functions among genes that were contributed to treatment explained variance. Fig S2. Principal component analysis for bulk RNAseq data.Canonical correlation of the relationship between the five first principal components and the particular groups of interest. “Treatment” corresponds to either iMB or yMB, “Timepoint” corresponds to the time of microbial transfer, “Reads” corresponds to a measure of library depth per sample, and “Genes” corresponds to a measure of library depth for genes present in all samples. Fig S3. Cell proportions as determined from single cell RNAseq data. A-B) Box plots of cell type proportions in epithelial cells (A) and immune cells (B) separated by treatment. iMB cell proportions are colored in blue and yMB in red. Cell proportions were calculated for each individual sample. Nonparametric t test statistics were used to compare the two treatments with corresponding p-value for each comparison. Fig S4. Antibiotic resistance gene counts do not differ among yMB and iMB. Counts of (A) total and (B) individual antibiotic resistance genes were determined in the metagenomic data from 72w and 120w samples of the yMB and iMB treatment groups. Table S1. Enriched KEGG pathways in fecal yMB and iMB metagenomes. Table S2. Enriched gene functions in bulk and single cell RNA sequencing data. The three worksheets contain (i) the legend with information on the terms and data and the enriched gene functions in (ii) bulk and (iii) single cell RNA sequencing data. Celltype = Cell type annotated for the single cell analysis. Signal = Gene down- or upregulated when compared between yMB and iMB interventition. ID = Gene ontology identification code. Description = Gene ontology description. We focused on Biological process. GeneRatio = Ratio of identified genes divided by all genes of the respective GO category. BgRatio = Ratio of the size of the geneset compared to all unique genes in entire collection. pvalue = Significance value. qvalue = FDR-adjusted p value. geneID = GeneIDs that contributed to the GO term. Count = Number of genes that contributed to the GO term. SYMBOL = Gene SYMBOL that contributed to the GO term. The table can be accessed via this permanent link: https://tinyurl.com/3az899ds. Table S3. Antibodies used for FACS analyses of isolated intestinal cells.

## Data Availability

The bulk and single-cell RNA sequencing data are deposited at NCBI Gene Expression Omnibus (https://www.ncbi.nlm.nih.gov/geo) under the accession numbers GSE272841 and GSE272842. The microbiome sequencing data are accessible through the European Nucleotide Archive (https://www.ebi.ac.uk/ena) under the accession number PRJEB77113.
