# Supplementary material for: Life-long microbiome rejuvenation improves intestinal barrier function and inflammaging in mice
Source: Microbiome. 2025 Apr 2;13:91. doi: 10.1186/s40168-025-02089-8 (PMC11963433; doi:10.1186/s40168-025-02089-8)
Supplement: Supplementary file 2 — Additional file 1. Fig S1. Bulk transcriptional adaptations to the microbiome rejuvenation in ileum tissue. Bulk RNA sequencing was performed on ileum tissue of 40- and 120-week-old yMB and iMB mice. A) Principal component plot. B) Genes which expression changes contributed to the observed variance attributed to timepoint or treatment model. C) Enriched functions among genes that were contributed to treatment explained variance. Fig S2. Principal component analysis for bulk RNAseq data.Canonical correlation of the relationship between the five first principal components and the particular groups of interest. “Treatment” corresponds to either iMB or yMB, “Timepoint” corresponds to the time of microbial transfer, “Reads” corresponds to a measure of library depth per sample, and “Genes” corresponds to a measure of library depth for genes present in all samples. Fig S3. Cell proportions as determined from single cell RNAseq data. A-B) Box plots of cell type proportions in epithelial cells (A) and immune cells (B) separated by treatment. iMB cell proportions are colored in blue and yMB in red. Cell proportions were calculated for each individual sample. Nonparametric t test statistics were used to compare the two treatments with corresponding p-value for each comparison. Fig S4. Antibiotic resistance gene counts do not differ among yMB and iMB. Counts of (A) total and (B) individual antibiotic resistance genes were determined in the metagenomic data from 72w and 120w samples of the yMB and iMB treatment groups. Table S1. Enriched KEGG pathways in fecal yMB and iMB metagenomes. Table S2. Enriched gene functions in bulk and single cell RNA sequencing data. The three worksheets contain (i) the legend with information on the terms and data and the enriched gene functions in (ii) bulk and (iii) single cell RNA sequencing data. Celltype = Cell type annotated for the single cell analysis. Signal = Gene down- or upregulated when compared between yMB and iMB interventition. ID = [file 40168_2025_2089_MOESM1_ESM.docx]

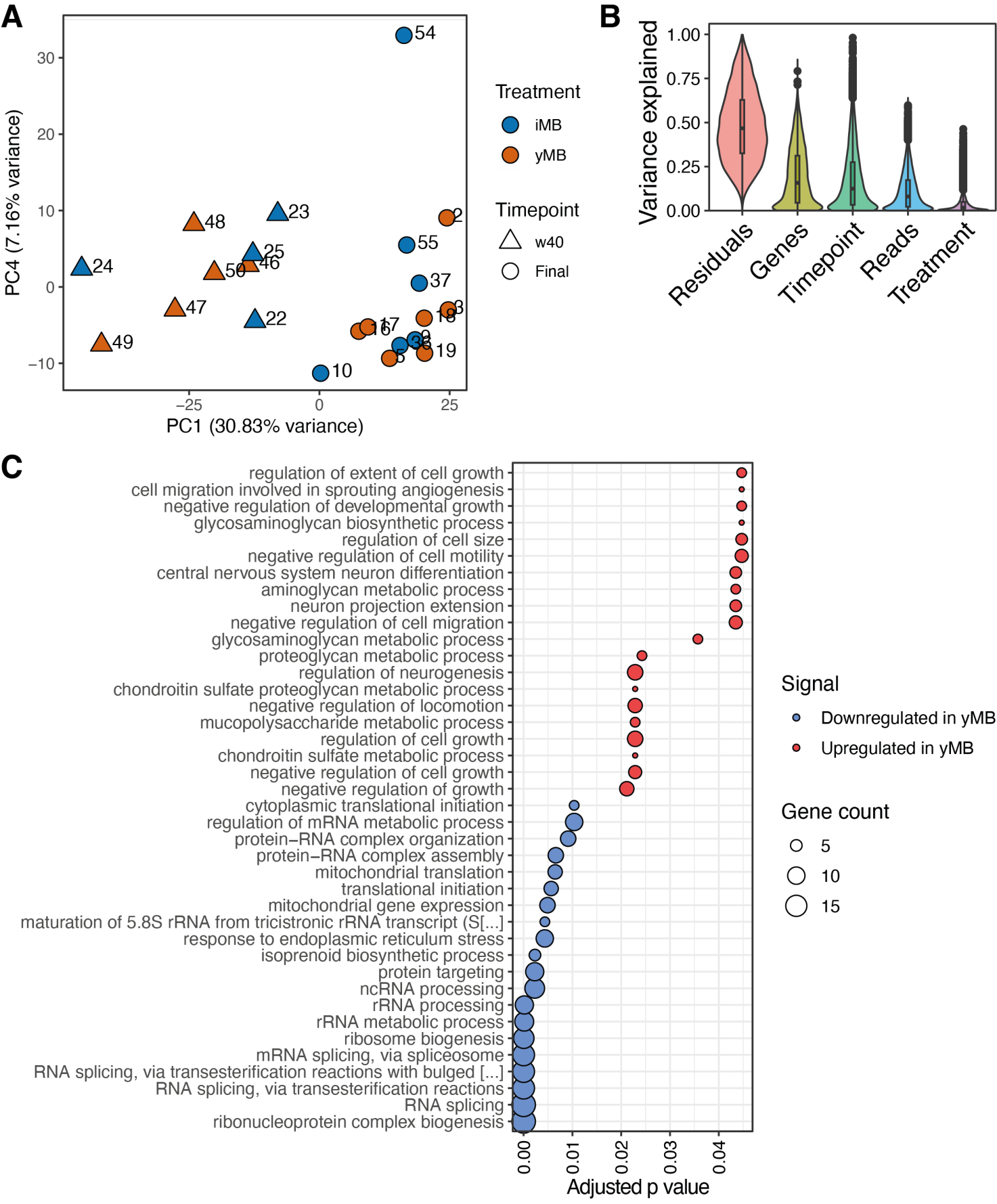


**Figure S1: Bulk transcriptional adaptations to the microbiome rejuvenation in ileum tissue.** Bulk RNA sequencing was performed on ileum tissue of 40- and 120-week-old yMB and iMB mice. **A)** Principal component plot. **B)** Genes which expression changes contributed to the observed variance attributed to timepoint or treatment model. **C)** Enriched functions among genes that were contributed to treatment explained variance.

**
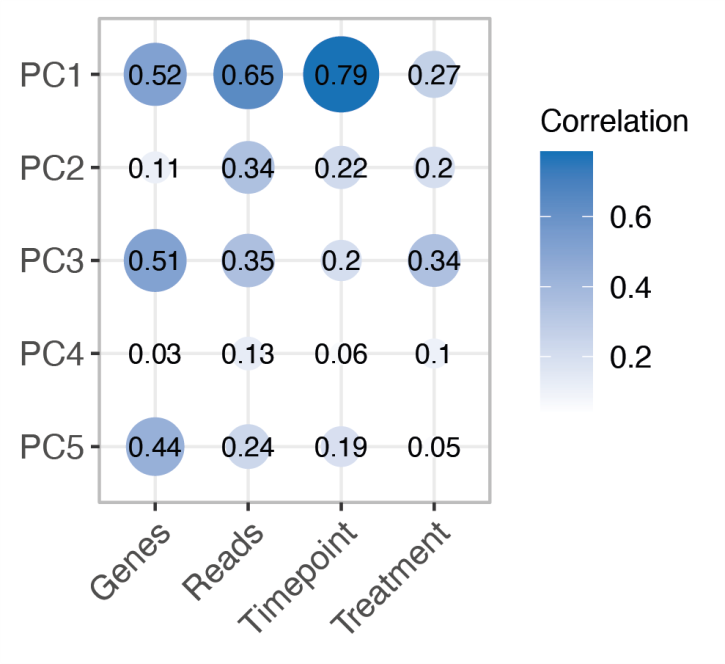
**

**Figure S2: Principal component analysis for bulk RNAseq data.** Canonical correlation of the relationship between the five first principal components and the particular groups of interest. “Treatment” corresponds to either iMB or yMB, “Timepoint” corresponds to the time of microbial transfer, “Reads” corresponds to a measure of library depth per sample, and “Genes” corresponds to a measure of library depth for genes present in all samples.


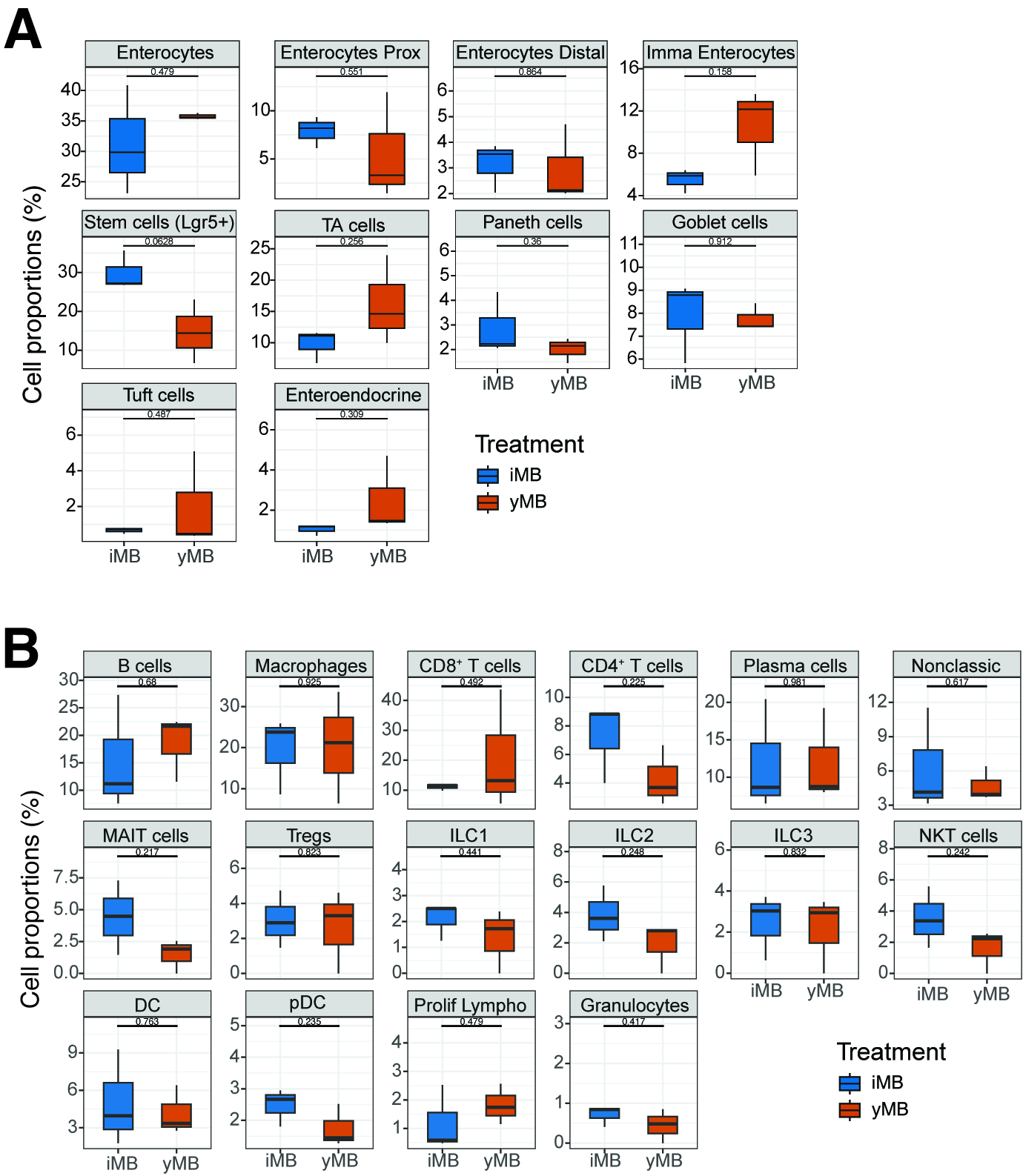


**Figure S3: Cell proportions as determined from single cell RNAseq data. A-B)** Box plots of cell type proportions in epithelial cells **(A)** and immune cells **(B)** separated by treatment. iMB cell proportions are colored in blue and yMB in red. Cell proportions were calculated for each individual sample. Nonparametric t test statistics were used to compare the two treatments with corresponding p-value for each comparison.


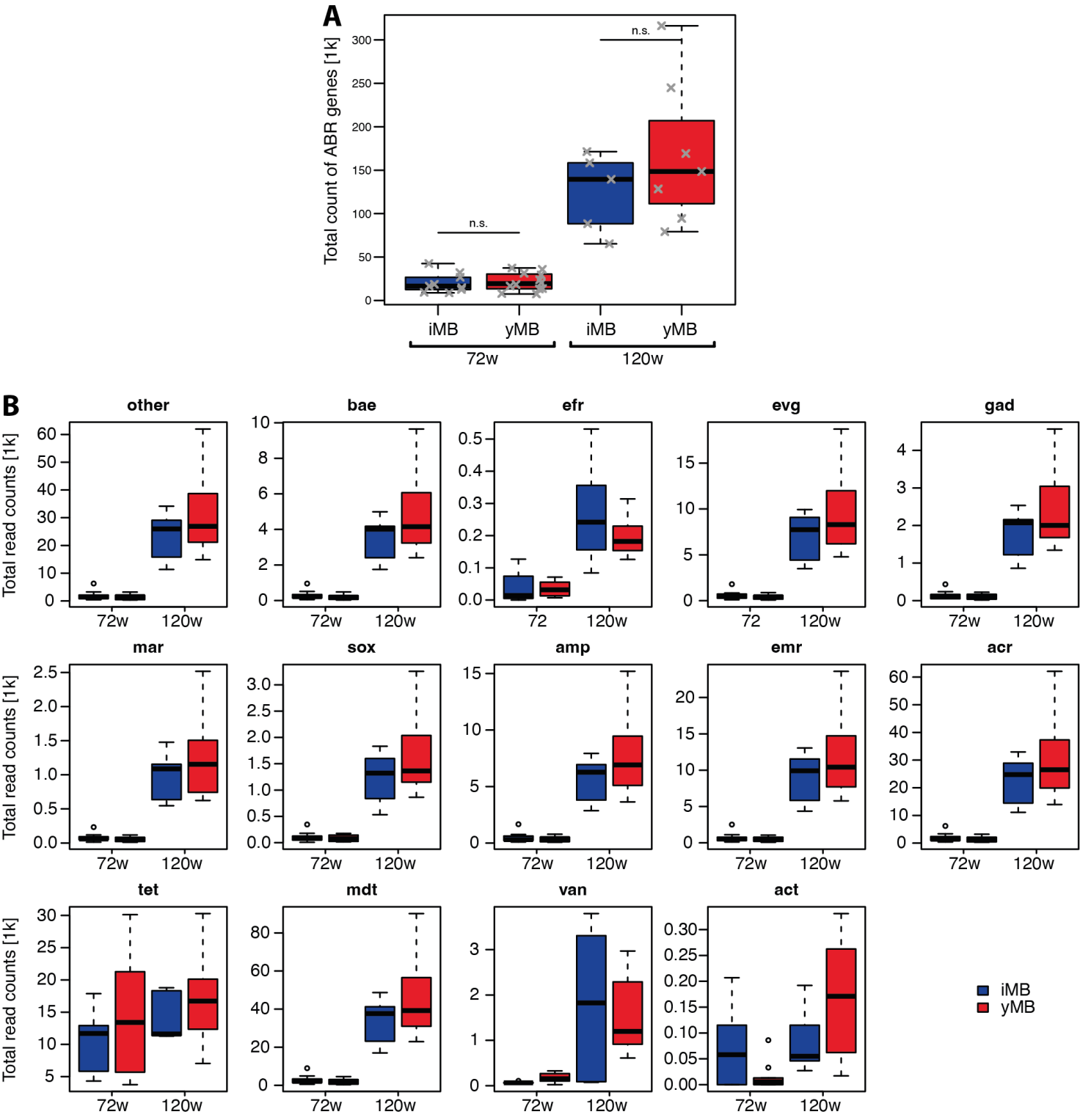


**Figure S4: Antibiotic resistance gene counts do not differ among yMB and iMB.** Counts of **(A)** total and **(B)** individual antibiotic resistance genes were determined in the metagenomic data from 72w and 120w samples of the yMB and iMB treatment groups.

**Table S1: Enriched KEGG pathways in fecal yMB and iMB metagenomes.**

| **Pathway description** | **ID** | **GeneRatio** | **BgRatio** | **p value** |
| --- | --- | --- | --- | --- |
| Antifolate resistance | ko01523 | 3/189 | 4/865 | 0.03453911 |
| Arginine biosynthesis | ko00220 | 7/189 | 16/865 | 0.0402238 |
| Atrazine degradation | ko00791 | 3/189 | 3/865 | 0.01030198 |
| Biosynthesis of amino acids | ko01230 | 24/189 | 60/865 | 0.00075241 |
| Biosynthesis of cofactors | ko01240 | 38/189 | 99/865 | 5.05E-05 |
| Biosynthesis of secondary metabolites | ko01110 | 57/189 | 203/865 | 0.01015892 |
| DNA replication | ko03030 | 4/189 | 6/865 | 0.0229027 |
| Homologous recombination | ko03440 | 10/189 | 14/865 | 8.84E-05 |
| Mismatch repair | ko03430 | 7/189 | 10/865 | 0.00141367 |
| Nicotinate and nicotinamide metabolism | ko00760 | 8/189 | 19/865 | 0.03647414 |
| Nucleotide excision repair | ko03420 | 4/189 | 5/865 | 0.00920413 |
| One carbon pool by folate | ko00670 | 8/189 | 12/865 | 0.00099776 |
| Oxidative phosphorylation | ko00190 | 11/189 | 27/865 | 0.01931251 |
| Peptidoglycan biosynthesis | ko00550 | 5/189 | 10/865 | 0.04563347 |
| Phenylalanine, tyrosine and tryptophan biosynthesis | ko00400 | 6/189 | 11/865 | 0.01737731 |
| Purine metabolism | ko00230 | 16/189 | 48/865 | 0.04015466 |
| Pyrimidine metabolism | ko00240 | 12/189 | 32/865 | 0.02964892 |
| Terpenoid backbone biosynthesis | ko00900 | 6/189 | 10/865 | 0.00962898 |
| Thiamine metabolism | ko00730 | 8/189 | 13/865 | 0.00211115 |

**Table S2: Enriched gene functions in bulk and single cell RNA sequencing data.** The three worksheets contain (i) the legend with information on the terms and data and the enriched gene functions in (ii) bulk and (iii) single cell RNA sequencing data. Celltype = Cell type annotated for the single cell analysis. Signal = Gene down- or upregulated when compared between yMB and iMB interventition. ID = Gene ontology identification code. Description = Gene ontology description. We focused on Biological process. GeneRatio = Ratio of identified genes divided by all genes of the respective GO category. BgRatio = Ratio of the size of the geneset compared to all unique genes in entire collection. pvalue = Significance value. qvalue = FDR-adjusted p value. geneID = GeneIDs that contributed to the GO term. Count = Number of genes that contributed to the GO term. SYMBOL = Gene SYMBOL that contributed to the GO term.

The table can be accessed via this permanent link: <https://tinyurl.com/3az899ds>

**Table S3: Antibodies used for FACS analyses of isolated intestinal cells.**

| **Type** | **Target protein** | **Fluorochrome** | **Dilution** | **Isotype** | **Company** | **Catalogue number** |
| --- | --- | --- | --- | --- | --- | --- |
| Target antibody | CD11b | PE | 1:100 | mono rat IgG2b | eBioscience | 12-0112 |
| Target antibody | CD11b | APC | 1:100 | mono rat IgG2bk | eBioscience | 17-0112 |
| Isotype control | CD11b | APC | 1:100 | mono rat IgG2bk | eBioscience | 17-4031 |
| Target antibody | CD11c | APC | 1:50 | Armenian Hamster IgG | eBioscience | 17-0114 |
| Isotype control | CD11c | APC | 1:50 | Armenian Hamster IgG | eBioscience | 17-4888 |
| Target antibody | CD19 | APC | 1:40 | rat IgG2a kappa | eBioscience | 17-0193-80 |
| Isotype control | CD19 | APC | 1:40 | rat IgG2a kappa | eBioscience | 17-4321 |
| Target antibody | CD25 | FITC | 1:4 | mono rat, IgG2b k | Immunotools | 22-150253 |
| Isotype control | CD25 | FITC | 1:4 | Rat IgG2b, k | Immunotools | 22225033 |
| Target antibody | CD4 | FITC | 1:6 | Rat IgG2b | Immunotools | 22150043 |
| Target antibody | CD4 | PE | 1:6 | mono rat IgG2b | Immunotools | 22150044 |
| Isotype control | CD4 | PE | 1:6 | rat IgG2b | Immunotools | 22225034 |
| Isotype control | CD8a | eFluor660 | 1:40 | rat IgG2a kappa | eBioscience | 50-4321 |
| Target antibody | CD95 | PE | 1:6 | Armenian Hamster IgG2 | BD | 554258 |
| Isotype control | CD95 | PE | 1:6 | Hamster IgG2 | BD | 553965 |
| Target antibody | EpCam | APC | 1:10 | rat IgG2aK | Biolegend | 118214 |
| Isotype control | EpCam | APC | 1:10 | rat IgG2aK | Biolegend | 400512 |
| Target antibody | Ly6G | FITC | 1:100 | rat, IgG2b kappa | eBioscience | 11-5931 |
| Isotype control | Ly6G | FITC | 1:100 | rat-IgG2b | eBioscience | 11-4031 |
| Isotype control | MHC I | FITC | 1:100 | mouse IgG2a, k | eBioscience | 11-4724 |
| Target antibody | MHCI | FITC | 1:100 | mouse IgG2a, k | eBioscience | 11-5999 |
| Target antibody | MHCII | PE | 1:100 | rat IgG2b | eBioscience | 12-5322 |
| Isotype control | MHCII | PE | 1:100 | rat IgG2b | eBioscience | 12-4031 |
